# Supplementary material for: Spin-current emission governed by nonlinear spin dynamics
Source: Sci Rep. 2015 Oct 16;5:15158. doi: 10.1038/srep15158 (PMC4607955; doi:10.1038/srep15158)
Supplement: Supplementary Information [file srep15158-s1.pdf]

Supplementary Materials for

Spin-current emission governed by nonlinear spin dynamics

Takaharu Tashiro, Saki Matsuura, Akiyo Nomura, Shun Watanabe, Keehoon Kang, Henning Sirringhaus, and Kazuya Ando,*

*To whom correspondence should be addressed; E-mail: ando@appi.keio.ac.jp

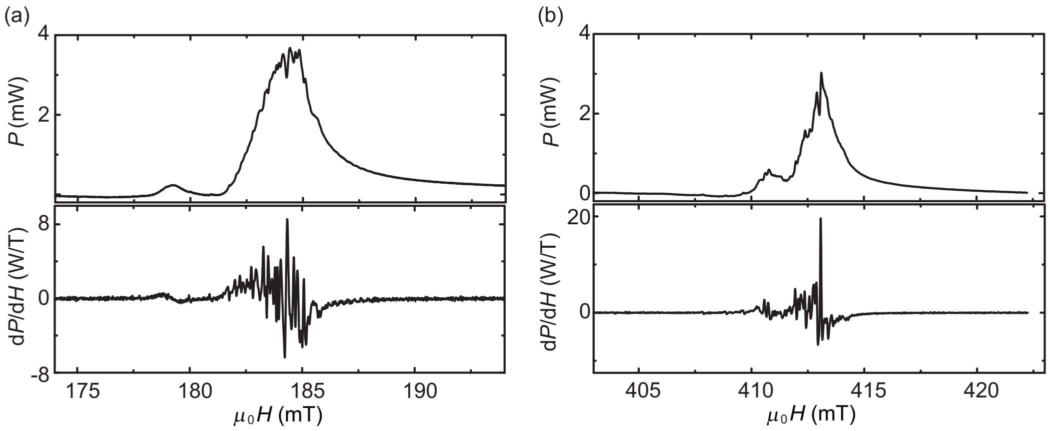

Figure 1: **Microwave absorption spectra.** (a) In-plane magnetic field (H) dependence of the microwave absorption P and dP/dH for the Pt/$Y_3Fe_5O_{12}$ film at f_0 = 7.6 GHz and P_{in} = 10 mW. (b) Out-of-plane H dependence of P and dP/dH for the Pt/$Y_3Fe_5O_{12}$ film. The absorption peak comprises multiple signals due to spin-wave modes (see the dP/dH spectrum). This makes it difficult to extract the linewidth of the ferromagnetic resonance mode.

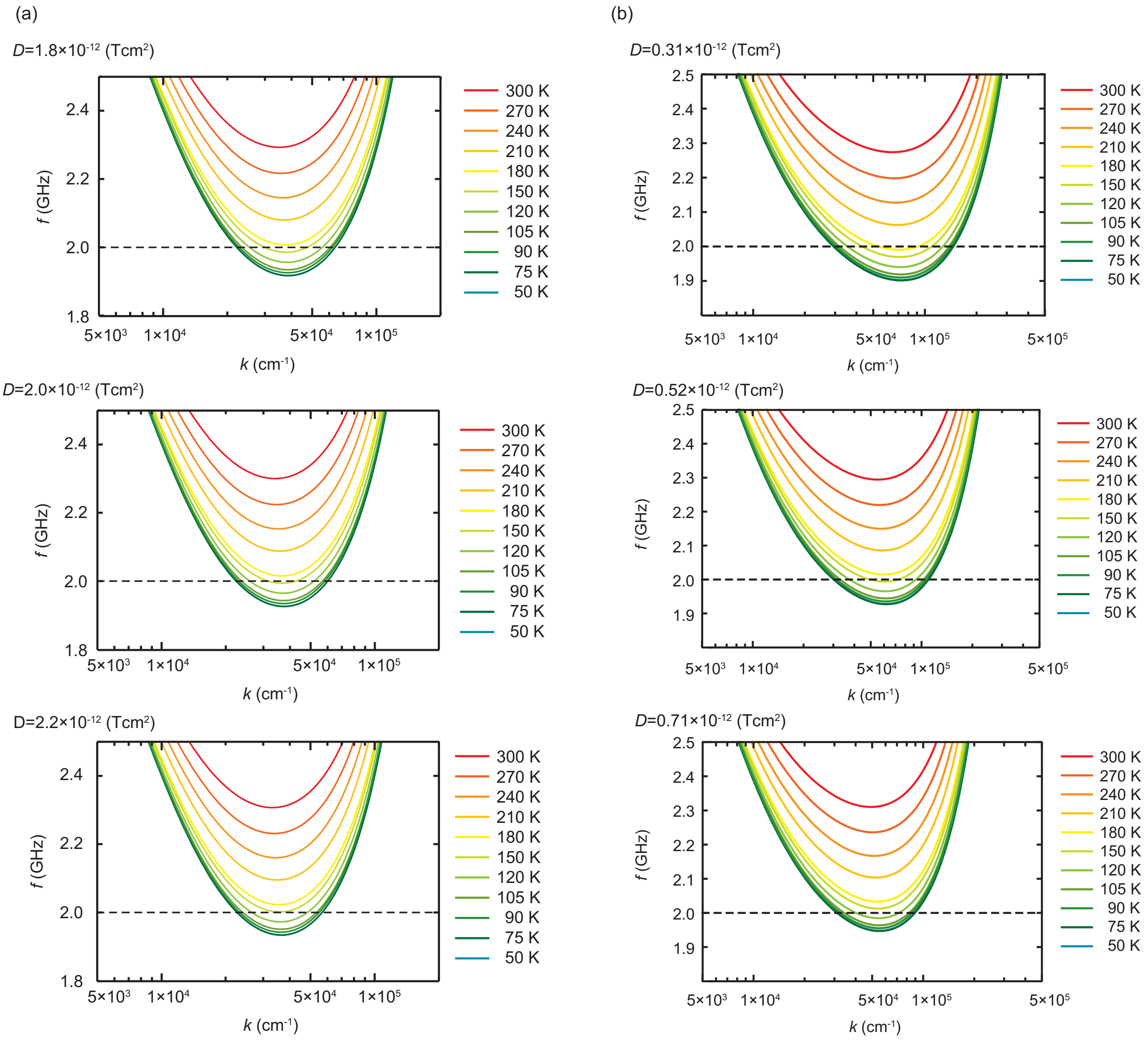

Figure 2: **Spin-wave dispersions for different boundary conditions.** (a) Spin-wave dispersions for the pinned surface spins condition for $D = 1.8 \times 10^{-12}$ Tcm^2, 2.0×10^{-12} Tcm^2, and 2.2×10^{-12} Tcm^2. (b) Spin-wave dispersions for the unpinned surface spins condition for $D = 0.31 \times 10^{-12}$ Tcm^2, 0.52×10^{-12} Tcm^2, and 0.71×10^{-12} Tcm^2. For the calculations, the exchange interaction constant D was assumed to be constant because of the negligibly small temperature dependence of D. The temperature-dependent saturation magnetization M_s used for the calculation was obtained from the microwave frequency dependence of the ferromagnetic resonance field at each temperature (see Fig. 3(c) in the main text).

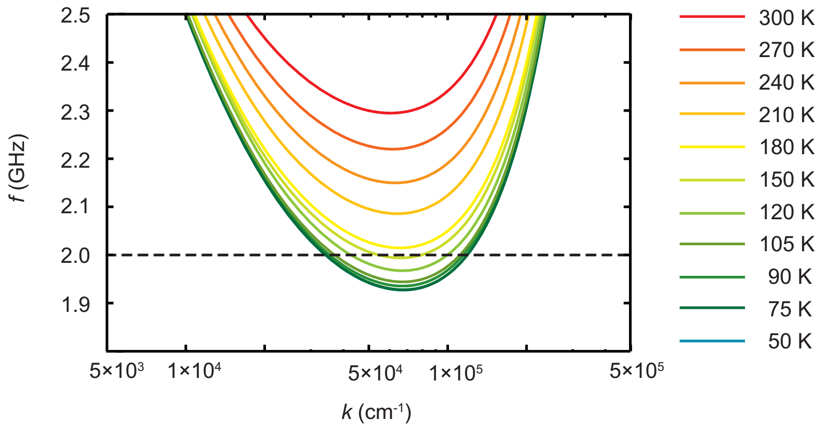

Figure 3: **Spin-wave dispersions for the Rado-Wertmann boundary condition.** Spin-wave dispersions for the Rado-Wertmann boundary condition[1] calculated with the surface pinning parameter[2] $d = 50 \times 10^6$ m^{-1}. The exchange interaction constant $D = 4.5 \times 10^{-13}$ Tcm^2 was used for the calculation. The temperature-dependent saturation magnetization M_s used for the calculation is shown in Fig. 3(c) in the main text.

[1] Kalinikos, B. A. & Slavin, A. N. Theory of dipole-exchange spin wave spectrum for ferromagnetic films with mixed exchange boundary conditions. *J. Phys. C* **19**, 7013 (1986).

[2] Xiao, J., Zhou, Y. & Bauer, G. E. Spin-wave excitation in magnetic insulator thin films by spin-transfer torque. In Wu, M. & Hoffmann, A. (eds.) *Recent Advances in Magnetic Insulators — From Spintronics to Microwave Applications*, vol. 64 of *Solid State Physics*, 29 – 51 (Academic Press, 2013).
